# Progress and Perspectives on Pyrite-Derived Materials Applied in Advanced Oxidation Processes for the Elimination of Emerging Contaminants from Wastewater

**DOI:** 10.3390/molecules30102194

**Published:** 2025-05-17

**Authors:** Jannat Javed, Yuting Zhou, Saad Ullah, Tianjiu Gao, Caiyun Yang, Ying Han, Hao Wu

**Affiliations:** 1 Hebei Key Laboratory of Heavy Metal Deep-Remediation in Water and Resource Reuse, Key Laboratory of Applied Chemistry, School of Environmental and Chemical Engineering, Yanshan University, Qinhuangdao 066004, China; jannatsaad27@yahoo.com (J.J.);; 2 School of International Education, Yanshan University, Qinhuangdao 066004, China; saadullah4992@yahoo.com; 3 State Key Laboratory of Metastable Materials Science and Technology, Key Laboratory of Microstructural Material Physics of Hebei Province, School of Science, Yanshan University, Qinhuangdao 066004, China; 4 Shenzhen Research Institute of Yanshan University, Shenzhen 518000, China

**Keywords:** pyrite-derived materials, advanced oxidation processes (AOPs), emerging contaminants (ECs), reactive oxygen species (ROS), wastewater treatment

## Abstract

Emerging contaminants (ECs) in wastewater threaten environmental and human health, while conventional methods often prove inadequate. This has driven increased concern among decision makers, justifying the need for innovative and effective treatment approaches. Pyrite-derived materials have attracted great interest in advanced oxidation processes (AOPs) as catalysts because of their unique Fe-S structure, ability to undergo redox cycling, and environmental friendliness. This review explores recent advances in pyrite-derived materials for AOP applications, focusing on their synthesis, catalytic mechanisms, and pollutant degradation. It examines how pyrite activates oxidants such as hydrogen peroxide (H_2_O_2_), peracetic acid (PAA), and peroxymonosulfate (PMS) can be used to generate reactive oxygen species (ROS). The role of multi-dimensional pyrite architectures (0D–3D) in enhancing charge transfer, catalytic activity, and recyclability is also discussed. Key challenges, including catalyst stability, industrial scalability, and Fe/S leaching, are addressed alongside potential solutions. Future directions include the integration of pyrite-based catalysts with hybrid materials, as well as green synthesis to improve practical applications. This review provides researchers and engineers with valuable insights into developing sustainable wastewater treatment technologies.

## 1. Introduction

A clean and secure water environment is essential for human well-being and is equally important to economic development. However, rapid growths in population and industrialization have significantly increased global water pollution. Industries such as petroleum, chemical engineering, textiles, dyeing, food production, pharmaceuticals, and metallurgy discharge large volumes of organic wastewater containing refractory and highly toxic pollutants, including polycyclic aromatic hydrocarbons, halogenated hydrocarbons, phthalates, pharmaceuticals, and insecticides. Many of these pollutions are now defined as emerging contaminants (ECs), which are synthetic or naturally occurring chemicals or microbial components that are not routinely monitored in the environment, but which have the potential to enter the environment and cause known or suspected adverse ecological and/or human health risks [1,2]. The list of ECs is extensive and includes a wide variety of chemicals, as listed in Table 1.

Even at low concentrations, ECs pose significant ecological and human health risks, including chronic toxicity to aquatic organisms, endocrine disruption, reproductive impairment, and developmental abnormalities. Some ECs can bio-accumulate and bio-magnify via food webs, increasing the risk to higher trophic levels, including humans. Additionally, ECs can undergo complex transformations in the environment, generating more toxic or persistent byproducts [13]. Addressing these issues requires effective wastewater treatment strategies, particularly for EC removal. However, conventional biological treatment methods used in municipal sewage plants are often inadequate, especially for ECs with low bioavailability. Given worsening environmental pollution and increasing water scarcity, there is an urgent need to develop innovative technologies for treating and recycling EC-containing organic wastewater.

Currently, advanced oxidation processes (AOPs) are highly effective for treating ECs in wastewater due to their powerful oxidation capabilities that enable the degradation of refractory organics. AOPs perform efficiently even under challenging environments, such as high temperatures and fluctuating pH levels, making them adaptable to diverse wastewater treatment scenarios, including those with complex and unpredictable pollutant profiles [14]. These processes rely on reactive oxygen species (ROS), such as hydroxyl radicals (·OH), which have a high oxidation potential of 2.80 V, significantly greater than conventional oxidants (e.g., dichromate, permanganate, and peroxide) [15]. The generation of ROS in AOPs, known as “activation”, can be achieved using various methods, including electrical input, light irradiation, ultrasound, or the utilization of catalysts [16].

Compared to other activation methods, the catalytic activation process by transition metals is easier to scale up and offers the advantages of low energy consumption and high catalytic activity. Commonly used transition metal-based catalysts in oxidant activation include cobalt, copper, manganese, iron, and composite materials containing one or more of them [17,18]. Although some transition metals, such as cobalt-/copper-based materials, exhibit high catalytic efficiency in degrading ECs via AOPs, they also release heavy metal ions during the reaction, posing environmental risks and limiting their practical applications. Therefore, preventing the leaching of active transition metal during AOPs remains a critical challenge that must be addressed [19].

Among commonly used transition metals, ferrous iron (Fe^2+^) based catalysts stand out due to their natural abundance, low cost, non-toxicity, ease of synthesis, excellent physicochemical and magnetic properties, and environmental compatibility. These attributes make Fe^2+^ one of the most effective catalysts in activating chemical oxidants to degrade ECs in water [20]. Although Fe^2+^ is more environmentally friendly than many other transition metals, its leaching reduces catalytic efficiency in AOPs. This is because free Fe^2+^ requires an acidic environment for optimal performance and, at high concentrations, can act as a scavenger, consuming reactive radicals and thereby diminishing overall system performance. Moreover, traditional Fe^2+^ catalysts often lead to the formation of iron sludge during reactions, resulting in significant Fe^2+^ loss and secondary pollution [21].

To address these challenges, researchers have developed solid catalysts, such as iron (hydro) oxides, iron minerals, iron alloys, and supported iron-based materials, for heterogeneous AOPs instead of directly using soluble Fe^2+^. However, a major limitation is the low reduction rate of Fe^3+^ in conventional Fenton reactions, which severely restricts the continuous degradation of contaminants and compromises system sustainability [22]. Interestingly, substantial works have shown that co-catalysts such as zero-valent metals, natural organic acids, single-atom catalysts, and metal sulfides could significantly accelerate Fe^3+^/Fe^2+^ cycle, breaking through rate-limiting steps and facilitating reactive species generation. Nevertheless, these co-catalysts are often sacrificed in the process, leading to quenching effects on reactive species and potentially causing secondary environmental pollution. Thus, the use of co-catalysts in peroxide activation processes is virtually a trade-off: while they enhance the rapid Fe^2+^ regeneration, they also result in reductant consumption and potential environmental risks. Seeking a material that inherently combines catalytic activity with self-regeneration and minimized environmental impact would be a promising advancement in sustainable water treatment technologies [23].

Pyrite, an abundant sulfide mineral naturally found in the Earth’s crust, was historically the primary source of industrial sulfur and sulfuric acid production. Over the past decades, extensive research has explored pyrite’s geochemical characteristics, mineral processing, and its role in acid mine drainage [24]. More recently, pyrite has shown significant potential in environmental remediation, including stabilizing hexavalent chromium, adsorbing arsenic, and promoting the abiotic dichlorination of chlorinated organics, hydrolytically removing microcrystals, and denitrifying nitrate [25].

Pyrite’s composition of Fe^2+^ and S_2_^2−^ ions allows for it to serve as a catalyst in AOPs for producing reactive species. The Fe^2+^ ions act as catalytic sites for activating peroxides, while highly reducible S_2_^2−^ species donate electrons to accelerate Fe (III) reduction to Fe (II), enhancing redox cycling [26]. Structurally, pyrite adopts a NaCl-like arrangement, where Fe^2+^ ions are enclosed within cubic face-centered S_2_^2−^ cages, minimizing rapid Fe^2+^ leaching during reactions. Although the Fe and S may be gradually depleted over prolonged catalytic use, their byproducts are relatively environmentally benign; for instance, water with up to 250 mg/L SO_4_^2−^ remains safe for use. With its distinctive crystalline traits and excellent electron transport qualities, pyrite is a desirable substance for AOPs and relevant environmental catalytic applications, playing a vital role in the field of wastewater treatment and environmental cleanup. Its resilience, coupled with eco-friendly byproducts, makes pyrite a valuable resource for advancing sustainable environmental catalysis [27].

In recent years, numerous successful experimental studies on pyrite-based catalysts have been conducted, and the research interest in this area remains strong. However, a comprehensive review on pyrite-derived environmental catalysis is still lacking. In response, this article presents an in-depth overview of the current methods for synthesizing pyrite and pyrite derived materials, explores their unique characteristics, and explains the underlying principles of pyrite-based environmental catalysis processes, as well as the potential development trends in this field. This review focuses on providing a basic knowledge base and providing useful references for researchers exploring pyrite-based environmental catalytic systems.

## 2. Pyrite and Its Role in Catalyzing Fenton-like Reactions

### 2.1. Pyrite’s General Properties, Risks, and Potential

**Figure 1:**
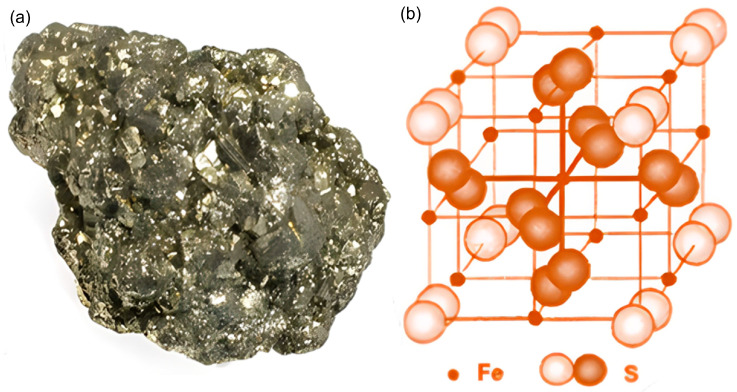
Pyrite mineral (**a**) and its crystal structure (**b**).

Pyrite (FeS_2_), also known as iron disulfide, is the sulfide mineral that is most prevalent, occurring in igneous, metamorphic, and sedimentary rocks, as shown in Figure 1a. In igneous rocks, it is commonly present as an accessory mineral and can form larger masses due to immiscible sulfide phases in the parent magma [28]. In metamorphic rocks, pyrite is often a product of contact metamorphism. While in hydrothermal systems, it is commonly associated with high-temperature conditions, although it can occasionally form under lower-temperature conditions [29].

Pyrite possesses a cubic crystal structure consisting of ferrous ions (Fe^2+^) and disulfide pairs (S_2_^2−^), which exhibit a robust lattice arrangement that imparts its metallic luster and high density [30]. The S_2_^2−^ unites consist of covalent boned sulfur atoms, stabilizing the cubic system and giving rise to pyrite’s distinctive morphologies. As shown in Figure 1b, the face-centered cubic sublattice of iron atoms makes up the unit cell of pyrite crystal, with S_2_^2−^ ions surrounded within it. Each Fe atom is octahedrally coordinated to six sulfur atoms, forming a slightly one-sided octahedron. Meanwhile, each S atom forms a tetrahedral connection with three Fe centers and one extra S atom [31].

Although pyrite is abundant and non-toxic, it can result in significant geological and environmental impacts. It is unstable under oxidizing conditions at the Earth’s surface, where factors such as pH and temperature strongly influence its decomposition. Under acidic conditions, pyrite readily dissolves and undergoes oxidation, releasing Fe^2+^ and sulfate ions (SO_4_^2−^) into the environment. This process contributes to environmental acidification, particularly in acid mine drainage scenarios [32]. Given this property, pyrite is usually commercially used to produce sulfur dioxide, which is subsequently used in sulfuric acid manufacturing. Additionally, pyrite oxidation is sufficiently exothermic, presenting hazards like spontaneous combustion in high-sulfur coal seams found in underground coal mines (commonly known as dust explosion). Techniques like buffer blasting and the application of different sealing or cladding agents are used to hermetically seal the mined-out portions to exclude oxygen in order to reduce these dangers [33]. Furthermore, pyrite in building materials, such as concrete, can undergo oxidation followed by reduction to form sulfides, leading to foul odors and the corrosion of metal wiring. Additionally, pyrite is essential to the geochemical cycling of sulfur and iron. Secondary minerals such as Jarosite (KFe_3_(SO_4_)_2_(OH)_6_) and goethite (FeOOH), may form as a result of its oxidation and dissolution under specific geochemical circumstances, which influence soil and water chemistry [34].

Despite the challenges associated with its redox characteristics, pyrite has encouraging promises in environmental restoration. Its surface properties enable the adsorption of heavy metals and other contaminants from aqueous solutions through interactions with various ions and molecules. Additionally, pyrite’s unique redox characteristics enhance its catalytic potential by allowing for it to function as both an electron donor and acceptor in diverse chemical reactions. This is capability is particularly valuable in AOPs, where catalytically activated peroxides to produce ROS to break down refractory pollutants in wastewater [35]. Recent advancements in pyrite-based composite materials, such pyrite–graphene hybrids, have further improved catalytic performance by stabilizing reactive intermediates and facilitating charge transfer [36]. These developments highlight pyrite’s versatility and its potential for environmental cleanup. To fully harness pyrite’s applications in AOPs, further research is needed to explore its material properties, catalytic mechanisms, recent innovations, and future prospects.

### 2.2. Pyrite-Catalyzed Fenton-like Reactions

The interaction between soluble Fe^2+^ and hydrogen peroxide (H_2_O_2_), leading to the generation of ·OH and other reactive oxidizing agents, is known as the Fenton reaction [37]. One successful method for getting around the drawbacks of the traditional Fenton reaction is adding a heterogeneous Fe^2+^ source, like pyrite, to the reaction solution, which requires a highly acidic condition to maintain the activity of Fe^2+^. Additionally, conventional Fenton processes are hindered by issues such as iron sludge formation, catalyst loss, and challenges in catalyst recovery, all of which severely restrict their practical application in real-world environmental scenarios [38].

A crucial step in utilizing FeS_2_ as a heterogeneous catalyst in AOPs is the emission of Fe^2+^ [39]. In aqueous environments, pyrite undergoes dissolution, liberating S_2_^2−^ and Fe^2+^, as represented by the following reaction (Equation (1)):

FeS_2_→Fe^2+^ + S_2_^2−^ (1)

When oxygen (O_2_) or other oxidants are present, the S_2_^2−^ undergo oxidation, ultimately forming sulfate ions (SO_4_^2−^). The transformation is described by Equation (2), shown as follows:

FeS_2_ + 7/2O_2_ + H_2_O→Fe^2+^ + 2SO_4_^2−^ + 2H+ (2)

While the formation of SO_4_^2−^ can affect the overall reaction kinetics and pollutant removal efficiency, the gradual release of Fe^2+^ from pyrite ensures sustained catalytic activity. This highlights pyrite’s dual role as both a source of Fe^2+^ and an electron donor, both necessary for advanced oxidation procedures to effectively degrade organic contaminants [40].

Pyrite has been confirmed as an effective activator for various peroxide oxidants, including hydrogen peroxide (H_2_O_2_), peracetic acid (PAA), and peroxymonosulfate (PMS) [41]. Through both Fe^2+^ and S species, pyrite-based Fenton-like catalysts enhance the activation of these oxidants, generating highly reactive radicals for pollutant degradation. Sulfur-mediated electron transfer not only improves reaction efficiency, but also prevents Fe hydroxide precipitation. Additionally, sulfur species facilitate Fe^3+^ reduction to Fe^2+^, stabilizing the redox cycle and producing secondary oxidants such as SO_4_^2−^ and polysulfides. This is because sulfur species such as S_2_^2−^ can be transformed into other sulfur-containing species during the peroxide activation process. Since the standard reduction potential of sulfur (S_2_^2−^/S^0^, −0.48 V) is much lower than that of iron (Fe (III)/Fe (II), 0.77 V), the regeneration of Fe (II) by S_2_^2−^ is thermodynamically favorable in the pyrite surface. Moreover, the consumed sulfur species can be converted to secondary oxidants such as SO_4_^2−^ and polysulfide to further degrade potential ECs [42].

In PMS-based systems, pyrite activates PMS to generate both sulfate radicals (SO_4_·^−^) and hydroxyl radicals (·OH). Compared to H_2_O_2_ systems, PMS reactions have a wider pH stability range (3–9), making them effective in near-neutral conditions. In PAA-based systems, pyrite catalyzes the PAA decomposition, forming acetyl radicals (CH_3_C(O)O·) and ·OH, enabling the selective degradation of specific pollutants [43]. Furthermore, sulfur species contribute to secondary oxidants such S^0^ and polysulfides, stabilizing the reaction and enhancing pollutant removal. Pyrite’s dual function of iron and sulfur chemistry allows for efficient degradation in both homogeneous and heterogeneous Fenton-like processes, making it a versatile catalyst various environmental applications (Table 2) [44]. 

#### 2.2.1. Pyrite-Derived H_2_O_2_ Activation

By generating ROS, pyrite effectively activates H_2_O_2_ in AOPs, facilitating the breakdown of organic contaminants. When added into a H_2_O_2_-containing system, pyrite dissolves, releasing S_2_^2−^ and Fe^2+^ into the aqueous phase (Equation (1)). Through the traditional Fenton reaction, the liberated Fe^2+^ activates H_2_O_2_, generating ·OH with a high oxidation potential of 2.8 V (Equation (3)) [45].

Fe^2+^ + H_2_O_2_→Fe^3+^ + ·OH + OH^−^ (3)

The ·OH generated from pyrite/H_2_O_2_ systems usually plays a vital role in the primary degradation of pollutants; for example, the reaction rate constant of pyrite/H_2_O_2_ derived ·OH was reported as 7.7 × 10^9^ M^−1^ s^−1^ and 2.2 × 10^10^ M^−1^ s^−1^ for tetracycline and salicylic acid [46].

Simultaneously, the S_2_^2−^ in pyrite enable the reduction of Fe^3+^ back to Fe^2+^, providing a continuous redox cycle with a high rate constant varying from 10^3^ to 10^6^ M^−1^ s^−1^ (Equation (4)) and ensuring ongoing radical generation [47].

Fe^3+^ + S_2_^2−^→Fe^2+^ + S_2_∙^−^ (4)

In addition to ·OH, the pyrite/H_2_O_2_ is capable of generating various ROS, such as superoxide radicals (O_2_·^−^), sulfate radicals (SO_4_·^−^), and peroxyhydroxyl radicals (HOO·), effectively improving the degradation of ECs.

(a) **Superoxide radical (O_2_·^−^):**

The reduction of Fe^3+^ by H_2_O_2_ or sulfur radicals (Equations (5) and (6)) [48]:

Fe^3+^ + H_2_O_2_→Fe^2+^ + HOO· + H^+^ (5)

HOO·↔O_2_·^−^ + H^+^ (6)

Although ·O_2_^−^ is highly reactive to organic pollutants such as ECs (rate constant varies from 10^−^^5^ to 10^−^^9^ M^−1^ s^−1^), its contribution in practical AOPs is usually lower than ·OH. This phenomenon is highly related to its stability in the conditions of AOPs, as it becomes more stable in alkaline conditions (t_1/2_ of 10–100 s) than neutral (t_1/2_ of 1–10 ms) and acidic conditions (t_1/2_ less than 1 μs).

(b) **Sulfate radicals (SO_4_·^−^):**

SO_4_·^−^ is formed from the oxidation of sulfur species in/dissolved from pyrite such as S_2_^2−^ by H_2_O_2_ or ·OH (Equation (7)) [49].

S_2_^2−^ + H_2_O_2_→2SO_4_^∙−^ + 2H + S_2_^2−^ + H_2_O_2_→2SO_4_·^−^ + 2H^+^ (7)

Overall, as a durable and efficient catalyst for H_2_O_2_ activation, pyrite produces a wide variety of ROS that aid in the effective breakdown of organic contaminants. However, the reaction between pyrite and H_2_O_2_ generates Fe^3+^, SO_4_^2−^, and H^+^, which causes the decline of the solution’s pH. Acidic conditions promote the dissolution of FeS_2_ and the generation of Fe^2+^. Conventional Fenton reactions utilize Fe^2+^ and H_2_O_2_ to produce ·OH, and the reaction between Fe^3+^ and H_2_O_2_ can transform Fe^3+^ back into Fe^2+^, but the low rate constant of this reaction limits the Fe^2+^/Fe^3+^ cycle, significantly influencing the sustainable activation of H_2_O_2_. The reduction of Fe^3+^ by pyrite is favorable for the Fe^2+^/Fe^3+^ redox cycle (Equations (4) and (8)). Its compatibility with various oxidants and capacity to sustain a continuous Fe^2+^/Fe^3+^ redox cycle make it a viable material for advanced oxidation processes in wastewater treatment [50].

(8) $${FeS}_{2}+{14Fe}^{3+}+{8H}_{2}O\to {15Fe}^{2+}+{2SO}_{4}^{2-}+16H^{+}$$

#### 2.2.2. PAA (C_2_H_4_O_3_) and Its Role in Pyrite-Derived AOPs

It has been shown that pyrite (FeS_2_) is a powerful catalyst for PAA activation, producing SO_4_·^−^, ·OH, and peracetic/organic radicals (R-O·), shown as Equation (9).

FeS_2_ + C_2_H_4_O_3_→Fe^2+^ + R-O· + SO_4_·^−^ (9)

**Figure 2:**
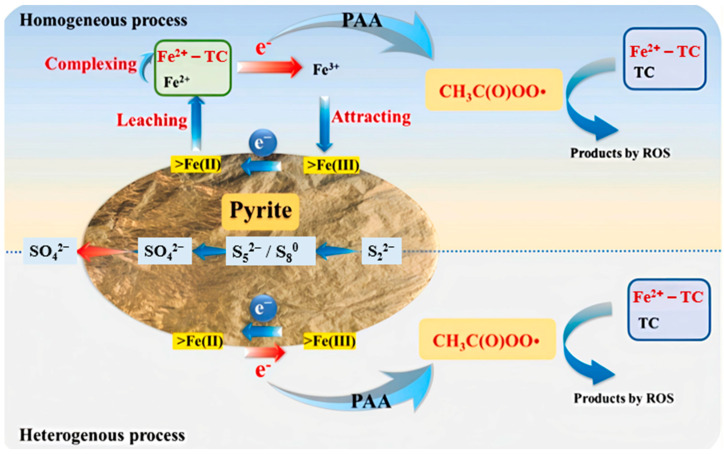
Methodology for TC removal from pyrite-induced PAA activation. Reprinted from Xing et al. [42]. Copyright 2022 Elsevier.

Pyrite’s sulfur species aid in electron transport, which maintains the Fe^2+^/Fe^3+^ redox cycle and increases PAA activation efficiency [51]. Taking the degradation of tetracycline as an example, Figure 2 illustrates the involvement of both homogeneous and heterogeneous routes in the pyrite-induced activation of PAA. In the homogeneous phase, Fe^2+^ leached from pyrite activates PAA, generating reactive radicals like CH_3_C(O)OO·, which degrade. The Fe^3+^ produced in this process is subsequently reduced back to Fe^2+^ by sulfur species on the pyrite surface. In the heterogeneous phase, PAA is adsorbed on surface Fe(II) sites, where electron transfer generates CH_3_C(O)OO· and converts Fe(II) to Fe(III). The sulfur species then reduce Fe(III) back to Fe(II), ensuring a sustained catalytic cycle.

Pyrite derived PAA activation is capable of generating ·OH and CH_3_C(O)OO· (Equation (10)). Reactions for PAA with Fe^2+^ possesses much lower activation energy (20.45 kcal mol^−^^1^ and 35.90 kcal mol^−^^1^ for ·OH and CH_3_C(O)OO· generation, respectively) than that of H_2_O_2_ (76.48 kcal mol^−^^1^) [42]. The generated CH_3_C(O)OO· can further react with O_2_ to form ·O_2_^−^ (Equation (11)).

CH_3_COOOH + Fe^2+^→CH_3_COO· + ·OH + Fe^3+^ (10)

CH_3_COO· + O_2_→CH_3_COOO·→CH_3_CO_2_H + O_2_·^−^ (11)

Additionally, PAA can facilitate the breakdown of persulfate into SO_4_·^−^, which has a powerful oxidizing ability that can attack organic contaminants in wastewater (Equation (12)) [52].

CH_3_COOOH + S_2_O_8_^2−^→CH_3_COO· + SO_4_·^−^ + SO_4_^2−^ (12)

#### 2.2.3. Activation of PMS by Pyrite

PMS (HSO_5_^−^) is a powerful oxidant used in AOPs due to its ability to generate highly reactive radicals. Pyrite can effectively activate PAA, producing SO_4_·^−^, ·OH, O_2_·^−^, and peroxymonosulfate radicals (SO_5_·^−^) [53].

Fe^2+^ in pyrite helps activate PMS by donating an electron, generating sulfate radicals (SO_4_·^−^) and peroxymonosulfate radicals (SO_5_·^−^) and sulfur species (S_2_^2−^) in pyrite enhance electron transfer, making radical formation more efficient (Table 3) [54].

SO_4_·^−^ is the typical ROS generated from PMS activation that can oxidize organic contaminants efficiently, shown as Equation (13) [55]:

Fe^2+^ + HSO^5−^→Fe^3+^ + SO_4_·^−^ + OH^−^ (13)

This reaction generates SO_4_·^−^, which has an oxidation potential of 2.5–3.1 V. In addition, SO_4_·^−^ can react with water to form ·OH (Equation (14)):

SO_4_·^−^ + H_2_O→·OH + HSO_4_^−^ (14)

Peroxymonosulfate radicals (SO_5_·^−^) are less reactive than SO_4_·^−^, but still contribute to the oxidation of water (Equation (15)):

SO_5_·^−^ + H_2_O→SO_4_·^−^ + H_2_O_2_ (15)

SO_5_·^−^ decomposes into SO_4_·^−^ and H_2_O_2_, which can further produce ·OH as a secondary ROS from SO_4_·^−^ and PMS breakdown, listed as Equation (16):

SO_4_·^−^ + H_2_O→^∙^OH + HSO_4_^−^ (16)

O_2_·^−^ are generated through Fe^3+^ cycling, shown as Equation (17). Although O_2_·^−^ is a weak ROS when compared with others, it plays a vital role in secondary oxidation and electron transfer [56].

Fe^3 +^ +O_2_→Fe^2 +^ +O_2_·^−^ (17)

When PMS is activated by pyrite, it follows these primary degradation pathways: (1) SO_4_·^−^ attacks organic contaminants (R-X), breaking them down into smaller intermediates [57]; (2) ·OH reacts with contaminants via hydrogen abstraction, leading to further degradation of organic matter into CO_2_, H_2_O, and mineralized end products; in addition (3) Fe^2+^ is regenerated through Fe^3+^ reacting with O_2_·^−^ (Equations (18)–(20)) (Table 4). This allows for the continuous PMS activation for sustained pollutant degradation [58].

SO_4_·^−^ + R−X→R· + SO_4_^2−^ + X^−^ (18)

·OH + R−H→R· + H_2_O (19)

Fe^3+^ + O_2_·^−^→Fe^2+^ + O_2_ (20)

where R represents an oxidized pollutant fragment.

## 3. Pure Pyrite Derived Catalysts and Their Applications

When practically using pyrite as a catalyst in AOPs, its dimensional structure, including 0D (nanoparticles), 1D (nanorods/nanowires), 2D (nanosheets), and 3D (hierarchical structures), significantly affect its catalytic activity, conductivity, and surface properties [59]. Optimizing the dimensional structure of pyrite-based materials can significantly improve pollutant degradation efficiency and promote more sustainable catalytic applications in wastewater treatment.

### 3.1. Zero-Dimensional Pyrite Materials

**Figure 3:**
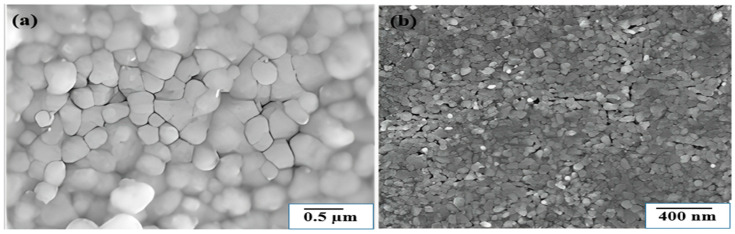
SEM pictures of sol–gel-produced FeS_2_ nanoparticles. (**a**) Sulfur annealing at 775 K for 4 h, (**b**) Films sulfurized from precursor films at 673 K for 10 h. Reprinted from Huang et al. [61]. Copyright 2010 Elsevier.

Zero-dimensional (0D) pyrite refers to nanoscale structures confined in all three dimensions, typically appearing as nanoparticles or quantum dots. This confinement induces the quantum effects, increases the surface-to-volume ratio, and enhances the catalytic and electrochemical properties. Due to these advantages, 0D pyrite is a material that shows promise for use in catalysis and environmental remediation [60]. Figure 3 summarizes some typical pyrite nanoparticles prepared using the sol–gel method. Annealing Fe(acac)_3_ ink on glass substrates coated with molybdenum (Figure 3a) and sulfonating FeO_3_ films on FTO substrates (Figure 3b) produced the nanoparticles, which showed formation with different morphologies. 

The effective application of 0D pyrite nanoparticles relies on efficient production methods. Various wet-chemical techniques, including precipitation, solvothermal, hydrothermal, and hot-injection methods, have been explored for their synthesis (Table 5) [62]. These techniques have successfully produced monodispersed FeS_2_ nanocrystals with high purity in the context of 0D pyrite nanoparticles [63]. In addition, surfactants like hexadecyltrimethylammonium bromide (CTAB) and gelatin have been shown to stabilize the nanoparticles, preventing aggregation and ensuring a uniform size distribution. Research indicates that 0D pyrites nanoparticles prepared from these above methods show a greater energy band gap of 0.73–1.6 eV than unstructured pyrite, highlighting the advantages in electron/photon response [64].

FeS_2_ materials can be synthesized using hydrothermal synthesis, the sol–gel technique, or the hot injection method. Hydrothermal synthesis is known for its ease of use and low cost, but requires precise temperature and pressure control, potentially causing scalability issues [66]. The sol–gel technique is ideal for producing pure and homogeneous FeS_2_ materials due to its superior homogeneity and compositional control, but has a long processing time and the need for post-synthesis heat treatment to eliminate solvents [67]. Furthermore, the hot injection method allows for fast synthesis of FeS_2_ nanocrystals with a limited size distribution. This approach is very efficient, but it involves high temperatures and precise reagent management, which might complicate the process and raise safety issues [68]. Each approach, while successful, has unique obstacles in terms of scalability, temperature control, and synthesis time that must be evaluated based on the desired application.

Due to 0D pyrite’s elemental composition and surface properties, it is widely employed to remove heavy metals, clean industrial effluents, and break down hazardous organic contaminants. Alam [69] reported that 0D pyrite nanoparticles effectively remove heavy metals from contaminated water, achieving adsorption rates of 92% for Pb^2+^ and 89% for As^3+^. This high efficiency is attributed to pyrite’s large surface area, which enhances its ability to capture heavy metal ions. Additionally, pyrite’s unique redox properties enable the transformation of hazardous high-valent metal ions into less toxic forms.

Zero-dimensional pyrite materials, particularly nanoparticles and quantum dots, have exhibited exceptional promise in AOPs. Sharma et al. [70] reported the utilization of 0D pyrite quantum dots to activate H_2_O_2_, generating O_2_·^−^ and ·OH, both of which are essential for the breakdown of persistent organic pollutants. As a result, up to 98% of organic dyes were removed in 60 min using this ROS-driven degrading process. In addition, the utilization of 0D pyrite in AOPs undergoes potential in microbial inactivation, antibiotic degradation, and waste-to-resource processes. A study investigated a mine waste derived 0D pyrite for persulfates activation in the degradation of tetracycline [71]. The results showed that 0D pyrite activated PMS more effectively than peroxydisulfate, achieving a high degradation rate of 98.3%, with up to 46% tetracycline (50 mg/L) being completely mineralized. Scavenging experiments indicated that both ·OH and SO_4_·^−^ were the main ROS, while SO_4_·^−^ was more dominant. Furthermore, an in vivo toxicity assessment indicated that the 0D pyrite/PMS system significantly decreased the nephrotoxicity (90%) and hepatotoxicity (85%) effects of tetracycline. Notably, no significant decline in catalytically performance was observed over five cycles. Overall, 0D pyrite is a non-toxic and clean catalyst in AOPs for ECs degradation and mineralization.

Despite the adaptability of 0D pyrite, challenges such as oxidation susceptibility and the formation of hazardous byproducts must be addressed to enable broader applications [72]. Otherwise, these issues could reduce ROS production and pose environmental risks. Key challenges include surface defects that lower catalytic performance, poor chemical stability due to oxidation, and the generation of hazardous sulfur species during degradation. Additionally, the synthesis of 0D pyrite is costly and challenging to scale, as it requires precise and controlled conditions. The aforementioned difficulties necessitate further in-depth investigations. Furthermore, commercial production remains limited, highlighting the need for studies focused on improving the stability and cost-effectiveness of 0D pyrite for practical applications [60]. To address these issues, researchers have shifted their focus to 1D and 2D pyrite structures, which offer enhanced stability, improved charge transfer, and prolonged ROS generation [73]. These advantages contribute to greater long-term efficiency in environmental remediation efforts.

### 3.2. One-Dimensional Pyrite Materials

**Figure 4:**
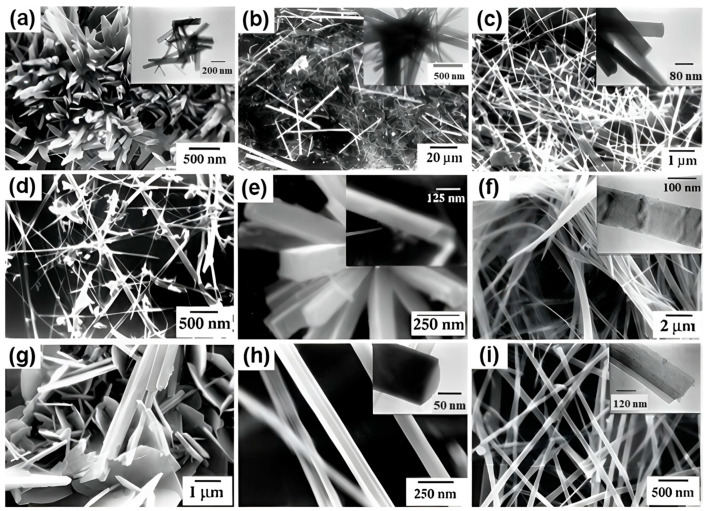
SEM and TEM images for various 1D FeS_2_ nanostructures synthesized from controlling iron sources, the molar concentration of precursors, and temperature. (**a**–**c**) Different iron sources, FeSO_4_, FeCl_3_, and Fe(NO_3_)_3_, respectively; (**d**–**g**) at 150 °C, 180 °C, 210 °C, and 230 °C; (**h**,**i**) half and double of the precursors, respectively. Reprinted from Kar and Chaudhuri [75]. Copyright 2004 Elsevier.

Materials including nanowires, nanorods, and nanotubes that have one dimension noticeably larger than the other two are referred to as 1D materials (Figure 4). Numerous techniques, including hydrothermal synthesis, vapor-phase growth, electrochemical deposition, and template-assisted techniques, can be used to create these structures. Iron sources, reaction time, and precursor concentration are some of the variables that might affect the microstructure of 1D pyrite. For instance, SEM and TEM pictures of 1D FeS_2_ nanostructures made with anodic aluminum oxide (AAO) templates are displayed in Figure 4. Using this method, Fe nanowires were electrodeposited into AAO pores initially, and the resulting FeS_2_ nanowires maintained their comparable sizes and morphologies following sulfurization. Furthermore, FeS_2_ nanowires and nanotubes were produced using a sol–gel technique in conjunction with AAO templating, illustrating the adaptability of this approach in accurately regulating nanostructure shape and size [74].

Various methods exist for synthesizing one-dimensional (1D) pyrite nanostructures like nanorods and nanowires (Table 6). The solvothermal approach is particularly effective due to its controlled reaction environment and flexibility in adjusting factors like solvent type, temperature, and reaction time. It offers strong crystallinity and good morphological control, but its use of organic solvents raises environmental concerns and is often high pressure and time consuming, restricting scalability [76]. The direct thermal sulfidation process uses high temperatures to react iron-based precursors with sulfur sources, resulting in FeS_2_. When suitable templates or growth-directing chemicals are used, this approach can produce crystalline 1D pyrite structures. It is reasonably easy and scalable, but it demands high temperatures, which may result in particle aggregation, less control over the aspect ratio, and smaller surface areas. The hydrothermal template-assisted technique is extremely effective for producing well-defined 1D structures by directing crystal development in certain orientations with soft or hard templates [77]. This approach allows for exact control over shape and size, resulting in high-aspect-ratio nanorods or wires with increased surface areas. However, it frequently includes sophisticated multi-step procedures, such as template preparation and removal, which can increase synthesis time and expense.

Unlike 0D nanoparticles, which often suffer from excessive grain boundaries and charge recombination, 1D FeS_2_ nanostructures offer continuous electron pathways that enhance electrical conductivity. Well-structured 1D pyrite FeS_2_ can address key challenges such as poor charge transport and instability, and, in some cases, even improve light adsorption. FeS_2_ nanorods and nanotubes exhibit high stability and electrochemical activity, making them well-suited for environmental catalysis and energy storage applications. Moreover, their elongated morphology enhances light trapping, improving efficiency in photovoltaics and light-driven Fenton reactions [81]. The pH of the reaction environment significantly affects the purity, morphology, and yield of FeS_2_ during synthesis. Acidic conditions (pH < 4) promote the production of pure FeS_2_ by reducing iron oxide impurities and increasing nucleation rates [82]. This frequently leads in the creation of spherical or agglomerated nanoparticles with great phase purity and quick crystal formation, as evidenced in the literature. At neutral pH (~7), the nucleation and growth processes are balanced, providing more control over particle size and shape. This pH range often produces 1D structures like nanorods and nanowires with high crystallinity and controllable dimensions [83]. Alkaline conditions (pH > 9) can cause iron hydroxides (Fe(OH)_3_) and sulfur species to precipitate and disrupt FeS_2_ production. These processes often create irregular or amorphous particles with low yield and restricted phase control. Maintaining an acidic to neutral pH range is ideal for creating well-defined FeS_2_ nanostructures with desired characteristics [84].

Given the unique structure of 1D pyrite, they are widely used in AOPs to generate ROS to degrade pollutants in both wastewater and atmosphere. Their effectiveness in AOPs is largely due to their ability to produce ROS, such as ·OH, which plays a critical role in breaking down industrial dyes, pharmaceuticals, and other organic contaminants. Their high surface area and electrical properties also make them promising for energy-related applications like hydrogen production and solar energy conversion [85].

Zhang et al. [86] reported that 1D pyrite nanowires significantly outperformed their 0D counterparts, achieving a 90% degradation efficiency of pharmaceutical contaminants in 80 min. This superior performance was attributed to enhanced charge transfer and a larger reactive surface area. Zeng et al. [87] demonstrated that visible light irradiation could enhance the oxidative performance of pure 1D pyrite in the degradation of p-nitro phenol. Under visible light, the complete oxidation time was reduced from 10 min to just 4 min, due to the activation of the Fe^3+^/Fe^2+^ redox cycle, which boosted ROS generation. DFT calculations indicated that this improvement was driven by the generation of valence band holes (h^+^), which readily react with other species under visible light exposure. Similarly to that of 0D pyrite, 1D pyrite has also been applied for heavy metal removal. It acts as an electron donor, reducing toxic metal ions such as Cr(VI) and As(III) into less harmful forms, while simultaneously generating ROS to further degrade associated pollutants [88]. In addition, the practical applicability of 1D pyrite materials can be further enhanced by integrating them into scalable reactor designs. In AOPs, these materials serve as efficient electron highways, facilitating rapid charge transfer and improved catalytic performance.

One-dimensional pyrite materials face several challenges that hinder their broader adoption, despite their promising performance. These include complex manufacturing processes, susceptibility to oxidation, and the potential generation of hazardous byproducts such as sulfur dioxide (SO_2_). The synthesis of 1D pyrite requires precise control over reaction conditions, making it costly and difficult to scale. As a result, its commercial availability remains limited [89].

To enhance their effectiveness in treating contaminants within complex wastewater matrices, researchers can optimize the surface-to-volume ratio of these materials. However, issues such as nanoparticle aggregation and mechanical fragility must also be overcome. Addressing these limitations, through the use of innovative synthesis approaches, can improve structural integrity and dispersibility, paving the way for more robust, scalable, and practical applications.

### 3.3. Two-Dimensional Pyrite Materials

Ultra-thin layered structures with distinct electrical characteristics and high surface-to-volume ratios are known as 2D pyrite materials. Their anisotropic charge transport and abundance of active edge sites contribute to significantly enhance catalytic activity. These materials can be synthesized using methods such as liquid-phase exfoliation, chemical vapor deposition (CVD), and hydrothermal synthesis. In advanced oxidation processes (AOPs), 2D pyrite has proven highly effective in degrading organic pollutants, activating hydrogen peroxide, and removing heavy metals. Additionally, they show strong potential in air purification and photocatalytic hydrogen production, further broadening their environmental and energy-related applications [90].

**Figure 5:**
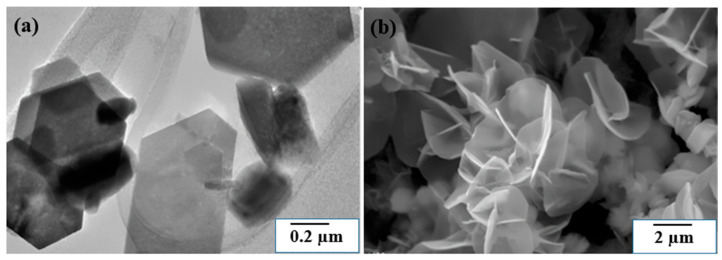
SEM images for (**a**) FeS_2_ nanoplates and (**b**) FeS_2_ nanosheets. Reprinted from Kirkeminde et al. [91] and Hu et al. [93]. Copyright 2012 and 2008 American Chemical Society.

Figure 5 illustrates the morphologies of synthesized 2D FeS_2_ nanostructures, including nanoplates and nanosheets. FeS_2_ nanoplates were produced using a reaction between Fe(CO)_5_ and an oleyl amine-coordinated elemental sulfur solution at temperatures of 180 °C or higher, followed by an aging period of over 180 min [91]. It was observed that increasing the reaction temperature significantly influenced lateral (planar) growth while having a minimal effect on the thickness of the nanoplates. At 240 °C, truncated hexagonal and triangular nanoplates with sizes ranging from 200 to 500 nm were obtained. In another study, FeS_2_ nanosheets were selectively synthesized via a one-step hydrothermal method. Using high-purity Fe foil (99.99%) and sulfur powder dissolved in deionized water, FeS_2_ nanosheets with diameters of approximately 2 μm and thicknesses around 30 nm were successfully produced after reacting at 160 °C for 12 h [92].

Two-dimensional pyrite materials demonstrate exceptional in AOPs due to their high surface area, excellent conductivity, and abundance of reactive sites. These properties enable them to effectively degrade persistent organic pollutants, activate peroxide oxidants to generate ROS, and remove heavy metals through a combination of adsorption, catalysis, and redox reactions. Tan et al. [94] reported that pyrite oxidation produces ROS with a high facet dependence. Different facet compositions of pyrites showed different efficiency in generating O_2_·^−^, H_2_O_2_, and ·OH. There was a considerable association between the ratio of the [2 1 0] facet and the 48 h OH· production rates, which varied by 3.1 times, from 11.7 ± 0.4 to 36.2 ± 0.6 nM/h. The main cause of this facet dependence in ROS productions is the variation in the kinetics (from 1.2 × 10^–4^ to 5.8 × 10^–4^/s) and surface electron-donating capabilities (2.2–8.6 mmol e^-^/g) of different faceted pyrites. These results demonstrate how important facet composition is in influencing the generation of ROS and the ensuing ROS-driven processes during the oxidation of iron minerals. Studies have demonstrated that 2D pyrite-based catalysts can degrade organic pollutants by more than 95% in 60 min, surpassing lower-dimensional pyrite, because of their high density of active sites and enhanced electron mobility [15]. Two-dimensional structures are more stable and recyclable than zero-dimensional and one-dimensional pyrite, which makes them ideal for long-term environmental cleanup applications [86]. Long-term environmental cleanup applications benefit greatly from the stability and recyclability that 2D structures provide over 0D and 1D pyrite.

Two-dimensional pyrite materials face several limitations, including structural instability, complex synthesis procedures, and high susceptibility to oxidation. Their ultrathin layered structure makes them prone to breaking and restacking, which can significantly reduce their catalytic efficiency. Despite these drawbacks, 2D pyrite nanostructures generally outperform 1D counterparts in photo-catalysis, solar energy conversion, and energy storage applications, owing to their larger surface area, superior light absorption, and enhanced ion transport pathways [95]. Additionally, their planar geometry allows for easy stacking and integration with other nanomaterials, offering opportunities for the development of advanced hybrid catalysts. However, the potential environmental risks posed by the leakage of hazardous byproducts must also be considered. Long-term stability and scalable production remain major obstacles. Future research should focus on improving exfoliation and stabilization strategies for use in photocatalytic and electrochemical AOPs, while also optimizing synthesis techniques to minimize restacking and preserve high reactivity.

### 3.4. Three-Dimensional Pyrite Materials

**Figure 6:**
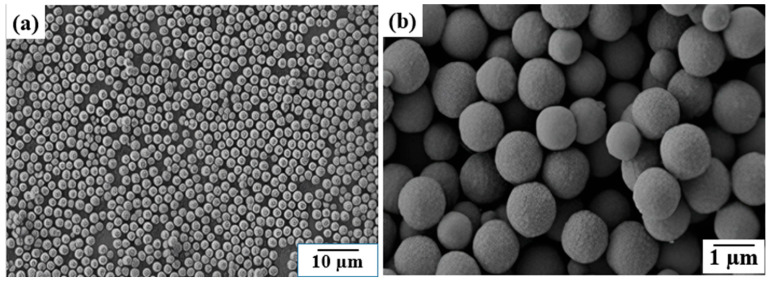
FeS_2_ microspherolites under microwave irradiation are shown in SEM pictures at low and high magnifications (**a**,**b**). Reprinted from Li et al. [97]. Copyright 2011 the Royal Society of Chemistry.

Three-dimensional pyrite materials are hierarchical architectures composed of interconnected nanoparticles, nanorods, or nanosheets. These structures provide a high surface area, improved charge transfer, and superior mass transport properties, making them highly suitable for catalytic and environmental applications. Common synthesis approaches include hydrothermal assembly, template-assisted synthesis, and electrodeposition [96]. Li et al. [97] reported the successful fabrication of FeS_2_ microspherolites using a microwave-assisted polyol method. Their study included reaction-time-dependent experiments to elucidate the aggregation mechanism. Additionally, FeS_2_ microspheres were synthesized via hydrothermal methods using polyvinylpyrrolidone (PVP) as a stabilizing agent. Figure 6 displays SEM images of FeS_2_ microspheroids synthesized using this method. The low-magnification images reveal the spherical morphology, while the high-magnification images reveal the surface texture and structure. These images demonstrate the successful formation of 3D FeS_2_ microspheroids with uniform sizes and shapes.

Three-dimensional pyrite materials are highly efficient in AOPs due to their high porosity, charge transfer properties, and interconnected structure [98]. Three-dimensional pyrite (FeS_2_) nanostructures, like microspheres or hierarchical architectures, outperform two-dimensional structures due to their enhanced stability, higher porosity, and improved mass transport [99].

In the degradation of organic pollutants, for example, 3D hierarchical pyrite structures have proven to have superior catalytic activity over lower-dimensional pyrite by promoting the generation of reactive oxygen species (ROS), which hasten the breakdown of dangerous pollutants [100]. Its improved adsorption and redox characteristics allowed for the removal of 99% of industrial emissions’ volatile organic compounds (VOCs), dramatically outperforming 2D and 1D pyrite structures in terms of long-term stability and reusability [101]. Zhang et al. [102] conducted a comparative study on the photo-Fenton degradation performance of sulfadiazine (SDZ) using 3D pyrite (FeS_2_) with three distinct morphologies: cube, octahedron, and sphere. The objective was to evaluate how morphological differences, particularly in terms of exposed crystal planes and sulfur (S) vacancy concentrations, influence catalytic efficiency. Among the tested structures, octahedral FeS_2_ exhibited the highest degradation (93.4%) and mineralization (82.3%) efficiencies. The enhanced performance was attributed to the synergistic effect of photo-catalysis in the Fenton reaction. Specifically, the (200) crystal planes exposed in the octahedral morphology possessed higher crystallographic energies, a larger specific surface area, and prominent sharp edges and corners, which collectively enhanced SDZ adsorption and facilitated Fe^2+^ release. Moreover, the narrowed band gap associated with these planes promoted the generation of photo-generated charge carriers. A higher density of S vacancies further contributed to increased active sites, extended carrier lifetimes, and improved photo-Fenton reactivity. Furthermore, it has been discovered that 3D foam-like pyrite structures effectively activate H_2_O_2_, resulting in a 120% increase in ROS formation when compared to 2D pyrite [103]. In addition to optimizing mass transfer and active site exposure, the linked framework of 3D pyrite materials also solves the aggregation and instability problems that are frequently seen in 0D, 1D, and 2D pyrite, making them extremely promising for environmentally friendly applications.

Three-dimensional pyrite materials, despite their advantages in catalysis and environmental remediation, face several challenges, including structural instability, complex synthesis processes, and susceptibility to oxidation [104]. These issues hinder their scalability and long-term practical application. To promote broader adoption, future research should focus on enhancing the structural stability of 3D pyrite and simplifying synthesis methods to allow for cost-effective large-scale production. Additionally, tailoring pore size and surface chemistry will be crucial for optimizing contaminant removal efficiency. Integrating 3D pyrite materials into intelligently designed reactors could pave the way for sustainable high-performance solutions in wastewater treatment and beyond.

A comprehensive overview of pyrite materials with various dimensional structures and their corresponding applications (including AOPs and non-AOPs) is presented in Table 7.

FeS_2_ nanostructures offer unique environmental benefits, such as removing nitrogen from wastewater and ammonia and nitrate in just four hours. Their high surface reactivity accelerates electron transfer, making them effective for redox-driven processes [111]. However, they tend to group together and are difficult to recover from. Future advancements could focus on surface modification or immobilization techniques. Nanorods and nanowires are examples of one-dimensional (1D) structures that provide improved electron transport and interaction with contaminants. They have shown that sophisticated oxidation techniques based on sulfate radicals can remove 90% of bisphenol A and that gas-phase detoxification of hydrogen sulfide can remove 95% of it [76]. Nevertheless, 1D structures may be mechanically weak and frequently have smaller surface areas. Their industrial feasibility may be enhanced by developments in flow-through reactor integration and structural reinforcing. Two-dimensional (2D) pyrite materials, such as nanosheets and thin films, are effective in surface-dependent processes [112]. Nanosheets can adsorb 90% of polystyrene microplastics within two hours due to their high surface areas and strong pollutant interactions. Thin films can achieve 92% degradation of pharmaceutical pollutants under solar illumination. However, 2D structures may face restacking issues and limited performance in low-light conditions. Three-dimensional (3D) morphologies like porous structures and hierarchical architectures are ideal for gas capture and heavy pollutant degradation [113]. Porous FeS_2_ frameworks have successfully converted up to 80% of CO_2_ into valuable chemicals, while hierarchical 3D pyrite successfully detoxified cyanide from mining effluents with a 97% success rate. However, these structures often require complex and expensive synthesis processes. Future approaches should prioritize scalable synthesis pathways and integration with continuous flow systems for industrial implementation.

The future of FeS_2_ morphology engineering is largely dependent on hybridization, stability optimization, and scalable fabrication methods that are adapted to the needs of individual applications. Each morphology has its own advantages and disadvantages, and the clever design of these morphologies will be essential to satisfying the expanding industrial and environmental demands.

## 4. Applications of Hybrid Pyrite

By integrating pyrite with sophisticated porous supports such as zeolites, biochar, and metal–organic frameworks (MOFs), hybrid pyrite-based materials have emerged as highly efficient catalysts for a range of catalysis and environmental applications (Table 8). These hybrid structures effectively overcome the inherent limitations of pure pyrite, such as oxidation susceptibility, leaching of Fe and S species, and structural instability, via enhancing material stability, catalytic performance, and recyclability. As a result, hybrid pyrite materials have found broad applications in photo-catalysis, energy storage, wastewater treatment, and the degradation of environmental pollutants [114].

Hybrid pyrite-based catalysts are promising in AOPs because they utilize the surface/elemental structure and/or electron transfer properties of the support materials to address the limitation caused by rapid oxidation and surface passivation in the pure form of pyrite, significantly increasing their performance and lifespan [118]. Zhou et al. [28] reported a study on the development of a pyrite-based catalyst by loading an S–Fe composite (primarily pyrite) onto waste-activated sludge-derived zeolitic materials, such as zeolite 4A and sodalite. This innovative design aimed to (1) disperse active Fe^2+^ within the pyrite structure, thereby broadening the applicable pH range and accelerating catalytic reaction rates; (2) stabilize Fe^2+^ using face-centered sulfur atoms, effectively reducing Fe leaching into the environment; and (3) exploit the redox properties of sulfur to regenerate active Fe^2+^, thereby extending the catalyst’s lifespan. The resulting pyrite–zeolite hybrid catalyst was employed to activate PAA for the treatment of various antibiotics and organic dyes in both real and simulated wastewater. The system achieved over 85% removal efficiency within 20 min at neutral pH (pH = 7). Compared to a control catalyst (Fe^2+^@zeolite without sulfur doping), the catalytic efficiency was enhanced by 26.7%. These findings underscore the advantages of integrating pyrite into zeolite supports, including improved catalytic activity across a broader pH range and enhanced durability [54].

Zhao et al. [119] reported a comparative study to evaluate the effectiveness of hybrid pyrite catalysts using activated carbon and biochar and carbon nanotubes as supporting materials, respectively, for the removal of ciprofloxacin. When the pseudo-first-order kinetic model of pyrite/activated carbon, pyrite/biochar, and pyrite/carbon nanotube with H_2_O_2_ was fitted to the synchronous experimental results, the reaction rates were 8.28, 3.40, and 3.37 times faster than those of pyrite alone under the experimental conditions. The synergistic effect of AOPs and the carbonaceous supports’ adsorption ability was credited with this notable performance improvement. Carbon materials facilitated the concentration of ciprofloxacin near the reactive sites while also promoting better dispersion of the pyrite particles, ultimately boosting the catalytic efficiency of the system. In addition, in pyrite-biochar material, S_2_^2−^, S_n_^2−^, and hydroxyl of biochar could act as electron donors involving in Fe(II)/Fe(III) cycle, promoting the oxidation of ECs and heavy metals [120]. The Fe-S active sites in pyrite facilitate the adsorption and reduction of toxic metal ions, such as arsenic (As^3+^), lead (Pb^2+^), mercury (Hg^2+^), and chromium (Cr^6+^) [86]. Hybrid structures, such as FeS_2_-loaded zeolites and FeS_2_-biochar composites, provide a high-surface-area scaffold, ensuring better metal capture and long-term stability. These materials can be used in both batch and continuous-flow water treatment systems, making them suitable for large-scale environmental applications [121,122].

Hybrid pyrite materials have also attracted considerable attention in the fields of photo-catalysis and solar energy conversion. Due to its strong light absorption and narrow bandgap (0.95–1.2 eV), pyrite is considered a promising candidate for solar-driven environmental remediation and hydrogen generation via photo-electrochemical (PEC) water splitting. However, the photocatalytic efficiency of pristine FeS_2_ is limited by issues such as photo-corrosion and rapid charge carrier recombination. These limitations can be effectively addressed through hybridization with materials such as TiO_2_, MOFs, or graphene oxide (GO), which significantly enhance charge separation, light absorption, and photocatalytic stability (Table 9). For example, FeS_2_–MOF composites have demonstrated excellent performance in visible light-driven wastewater treatment, while FeS_2_–TiO_2_ heterojunctions have shown improved solar-to-hydrogen conversion efficiency [60].

Moreover, the stability of catalysis is a vital factor in evaluating the practical application potential of pyrite-based materials. Metal leaching not only diminishes the catalytic activity of a material, but also poses significant risks of secondary environmental pollutions. Xing et al. [42] report the application of utilizing pyrite to activate PAA for tetracycline treatment. After three running cycles, although the observed reaction rate constant decreases from 0.123 to 0.792 min^−^^1^, the removal performance of tetracycline still reaches more than 90%. No significant changes were observed from their XRD results of pristine and used pyrite, indicating the crystal stability of pyrite. FTIR further exhibited that the decrease in catalytically reaction rate is only caused by pyrite surface passivation. Compared to common iron-based AOP catalysts, such as Fe particles, Fe-based MOFs, Fe-carbon, and Fe-zeolite, the S-Fe interaction in pyrite demonstrated competitive Fe fixation performance (Table 10).

All things considered, hybrid pyrite-based materials represent a significant advancement in the realms of green catalysis, renewable energy, and sustainable catalysis. By integrating pyrite with functional support materials, pyrite’s intrinsic limitations, such as oxidation susceptibility and structural instability, have been successfully addressed. To further expand the practical applications of these hybrid materials, future research should focus on optimizing material design, improving scalable synthesis methods, and facilitating industrial-scale implementation.

## 5. Conclusions

Pyrite-derived materials have revealed promise in the degradation of newly discovered pollutants in wastewater, which represents a major advancement in environmental remediation methods. Their unique Fe–S structure, ability to catalyze peroxide activation, and involvement in redox cycling position them as viable alternatives to conventional iron-based catalysts. However, challenges remain, including precise structural control, Fe and S leaching, and limited long-term stability. Future research should prioritize the development of hybrid pyrite-based composites, such as FeS_2_–graphene, FeS_2_–MOFs, and FeS_2_–TiO_2_, designed to enhance catalytic activity, stability, and reusability. Additionally, exploring multi-dimensional pyrite architectures (0D, 1D, 2D, and 3D) is essential to optimizing surface area, charge separation, and pollutant degradation efficiency.

To translate laboratory advances into real-world applications, pyrite-based AOPs must be adapted for integration into large-scale wastewater treatment systems. This includes investigating eco-friendly and scalable synthesis methods to ensure the sustainability and environmental safety of these materials. Moreover, the incorporation of machine learning and computational modeling could accelerate the rational design and performance prediction of next-generation pyrite catalysts. With their tunable properties and broad applicability in pollutant degradation, heavy metal remediation, and energy conversion, pyrite-based materials stand out as versatile and effective catalysts for future green technologies.

The practical optimization of pyrite-based advanced oxidation processes (AOPs) can be significantly accelerated through the integration of AI-driven catalyst design, computational modeling, and environmentally friendly synthesis strategies. These advanced tools offer the potential to predict material behavior, fine-tune structural parameters, and develop scalable fabrication techniques with reduced environmental impacts. By addressing the current limitations—such as instability, leaching, and structural control—while leveraging cutting-edge technologies, pyrite-based materials are well-positioned to make a substantial contribution to sustainable environmental remediation and energy storage systems. Ultimately, such innovations could pave the way for the widespread implementation of pyrite-derived catalysts in real-world applications.

## Figures and Tables

**Table 1:** ECs, their classification, and exempli gratia.

Classification	Exempli Gratia	Ref.
Pesticides	Glyphosate and atrazine	[3]
Pharmaceuticals	Diclofenac, ibuprofen, antibiotics, and hormones	[4]
Licit and illicit drugs	Caffeine, cocaine, and amphetamines	[5]
Preservatives	Parabens and triclosan	[6]
Personal care products	Sunscreens and UV filters	[7]
Surfactants, cleaning products, industrial formulations and chemicals	Bisphenol A and chlorinated solvents	[8]
Food additives and packaging	Phthalates and plasticizers	[9]
Polycyclic aromatic hydrocarbons, polychlorinated biphenyls, halogenated polycyclic aromatic hydrocarbons, polychlorinated naphthalene, dioxins, hexachloro-1,3-butadiene, polyhalogenated carbazoles, and environmentally persistent free radicals	Naphthalene, Fluoranthene Aroclor 1254, PCB-77 Brominated fluoranthenes, Fluorinated pyrenes Tetrachloronaphthalene, Hexachloronaphthalene Tetrabromocarbazole, Hexachlorocarbazole	[10]
Bromine-containing flame retardants, perfluorinated compounds and perfluorinated alkyl substances, brominated dioxins	Polybrominated diphenyl ethers (PBDEs) Perfluorooctanoic acid (PFOA) Hexabromodibenzofuran (HBDF)	[11]
Antibiotic-resistant pathogenic bacteria	*Escherichia coli* producing extended-spectrum β-lactamase	[12]
Other pollutants	Alkylphenols, metalloids, radionuclides, rare earth elements, nanomaterials, nanoparticles, microplastics, bioterrorism and sabotage agents, indoor pollutants, and pathogens	[2]

**Table 2:** Overview and contrast of homogeneous and pyrite derived heterogeneous Fenton flow.

Distinction	Homogenous Fenton Reaction	Pyrite Derived Heterogenous Fenton-like Reaction
Mechanism	Fe^2+^ + H_2_O_2_→Fe^3+^ + ·OH + OH^−^	Fe^3+^ + S_2_^2−^→Fe^2+^ + S_2_·^−^ Fe^2+^ + H_2_O_2_→Fe^3+^ + ·OH + OH^−^
pH	2.8–3.0	Wide Range
Reaction pathway with hydrogen peroxide/peroxide oxidants	Reaction of soluble Fe^2+^ with H_2_O_2_	Reaction of in situ Fe^2+^ in pyrite or leached Fe^2+^ from the catalyst with peroxide oxidants
Iron regeneration	Impossible	Redox derived Fe^2+^ regeneration and prolonged catalyst lifespan

**Table 3:** Activation of peroxymonosulfate (SO_5_·^−^) by pyrite.

Parameter	Details
Catalyst	Pyrite
Oxidant	HSO_5_^−^
Main Reactive Species Generated	Sulfate radicals (SO_4_·^−^), hydroxyl radicals (·OH), peroxymonosulfate radicals (SO_5_·^−^), and superoxide radicals (O_2_·^−^).
Role of Iron Species	Pyrite releases Fe^2+^, which activates PMS to generate radicals: Fe^2+^ + HSO^5−^→Fe^3+^ + SO_4_·^−^ + OH^−^ Fe^3+^ is reduced back to Fe^2+^, ensuring continuous redox cycling and sustained catalytic activity.
Degradation Efficiency	Highly efficient degradation of organic pollutants, including antibiotics, dyes, and industrial chemicals. Achieves up to 85–90% removal under optimized conditions.
Stability of Pyrite	Pyrite maintains structural integrity and catalytic efficiency over multiple reaction cycles. Sulfur species (S_2_^2−^) aid electron transfer, further stabilizing radical formation.
Applications	Waste water treatment, particularly for the degradation of persistent organic pollutants.
pH Influence	Works in a wide pH range (3–8), but acidic conditions (pH 3–5) enhance Fe^2+^ regeneration, maximizing radical production.

**Table 4:** Representative ROS generated from pyrite-derived systems and their characteristics.

ROS	Redox Potential (E)	Selectivity	Preferred pH Range	Main Reaction Mechanism	Target Pollutants/Transformation Pathway
·OH	~2.8 V	Non-selective	Acidic (~3–5)	H-abstraction, electron transfer, hydroxylation	Broad range: pharmaceuticals, dyes, organic acids; often leads to mineralization
SO_4_·^−^	2.5–3.1 V	Moderately selective	3–9	Electron transfer, H-abstraction	Electron-rich organics: phenols, antibiotics, EDCs
CH_3_COO·	~1.2–1.4 V	Highly selective (electrophilic)	3–7	Electrophilic attack, substitution	Electron-rich aromatics, halogenated compounds
SO_5_·^−^ (PMS radical)	~1.1 V	Weak oxidant	Variable	Oxygen transfer, precursor to SO_4_·^−^	Secondary oxidant; promotes slow oxidation or initiates SO_4_·^−^ generation

**Table 5:** Typical synthesis methods for 0D FeS_2_ materials.

Technique	Description	Key Features	Ref.
Hydrothermal synthesis	Involves the reaction of iron and sulfur precursors in an aqueous solution under high temperature and pressure in a sealed autoclave.	Produces highly crystalline nanoparticles. Environmentally friendly (uses water as solvent). Tunable size and morphology by adjusting temperature, pressure, and reaction time.	[64]
Sol–gel Technique	A low-temperature (≤100 °C) wet-chemical process where nano structures form through polymerization and gelation.	Low-temperature synthesis for FeS_2_ nanoparticles. Controlled polymerization is required.	[65]
Hot injection method	A high-temperature technique where a sulfur precursor is injected into an iron precursor under a protective atmosphere, forming FeS_2_ nanocrystals.	Produces high-quality single-crystalline FeS_2_ nanoparticles which could be used in photovoltaics and catalysis. Requires post-processing (washing, centrifugation, sintering at 540 °C for 4 h)	[60]

**Table 6:** Synthesis methods of 1D pyrite material.

Method	Iron Source	Sulfur Source	Reaction Conditions	Morphology	Ref.
Solvothermal	FeSO_4_·7H_2_O, FeCl_3_, Fe(NO_3_)_3_·9H_2_O	Thiourea (NH_2_CSNH_2_)	Ethylene diamine (EDA), 12 h at varying temperatures	Nanorods, nanowires	[78]
Direct thermal sulfidation	FeCl_2_, FeBr_2_	Sulfur vapor	425 °C, controlled sulfur super saturation	Nanorods, nanobelts, nanoplates	[79]
Hydrothermal template approach	ZnO nanorods (precursor)	Fe(NO_3_)_3_ and sulfur	350 °C for 3 h	Nanorod arrays	[80]

**Table 7:** Typical applications of pyrite-based catalysts for pollutant removal.

Type of Pyrite	Application	Performance Achieved	Main Findings	Ref.
0D Pyrite (nanoparticles)	Nitrogen removal in wastewater treatment.	Removed 85% of ammonia (NH_3_) and nitrate (NO_3_^−^) in 4 h.	Pyrite nanoparticles facilitated electron transfer, enabling rapid nitrogen conversion.	[44]
1D Pyrite (nanorods)	Sulfate radical-based AOP.	Achieved 90% removal of bisphenol A (BPA) in wastewater.	Nanorods exhibited enhanced sulfate radical activation, leading to superior degradation.	[105]
1D Pyrite (nanowires)	Gas-phase removal of hydrogen sulfide (H_2_S).	Removed 95% of H_2_S from industrial gas streams.	1D Pyrite acted as a sulfur scavenger, oxidizing toxic H_2_S into environmentally safe forms.	[106]
2D Pyrite (nanosheets)	Removal of microplastics from water.	Adsorbed 90% of polystyrene microplastics within 2 h.	2D Pyrite nanosheets provided high surface area for micro plastic entrapment and degradation.	[107]
2D Pyrite (thin films)	Photothermal degradation of organic pollutants.	Achieved 92% degradation of pharmaceutical pollutants under sunlight.	Pyrite thin films enhanced solar energy absorption, generating localized heat and ROS for effective pollutant breakdown.	[108]
3D Pyrite (porous structures)	CO_2_ capture and conversion.	Converted 80% of CO_2_ into carbonates and formic acid.	3D Pyrite structures improved CO_2_ adsorption, facilitating catalytic conversion.	[109]
3D Pyrite (hierarchical structures)	Detoxification of cyanide from mining waste.	Decomposed 97% of cyanide (CN^−^) in mining effluents.	3D hierarchical pyrite provided active sites for rapid cyanide degradation.	[110]

**Table 8:** Applications of hybrid pyrite-based materials.

Material	Application	Key Functions	Ref.
Zeolites	Water purification and wastewater treatment.	- Removes heavy metals (Pb, Cd, Cr, Ni) through ion exchange, adsorption and redox. - Eliminates ammonium (NH_4^+^_), nitrates (NO_3_^−^), and sulfates (SO_4_^2−^) from water. - Improves sedimentation and oxygen consumption in sewage treatment.	[115]
Biochar	Heavy metal adsorption and organic pollutant degradation.	- Adsorbs toxic metals like Pb, Cd, As, and Zn from contaminated water and soil. - Enhances persulfate activation for the breakdown of persistent organic pollutants.	[116]
Metal–organic frameworks (MOFs)	Pollutant removal, gas storage, and catalysis.	- Adsorbs and degrades organic pollutants and pharmaceutical residues. - Enhances heterogeneous catalysis for photocatalysis, hydrogen evolution, and AOPs.	[117]

**Table 9:** Advantages of hybrid materials.

Hybrid Material	Advantages	Ref.
FeS_2_-Graphene	Improved electronic conductivity, pollutant adsorption, and catalytic activity.	[123]
FeS_2_-TiO_2_	Enhanced photocatalytic efficiency under visible light.	[124]
FeS_2_-MoS_2_	High hydrogen evolution reaction (HER) efficiency.	[125]
FeS_2_-Metal–Organic Frameworks (MOFs)	Increased surface area and selective adsorption properties.	[126]

**Table 10:** Comparison of leaching quantities of current Fe-based catalysts.

Catalyst	Fe Stabilization Strategy	System	Leached Fe	Ref.
Residue Fe dust	None	pH = 7 H_2_O_2_	274.4 mg L^−1^	[127]
Fe oxide-SAPO-34	Fe oxide encapsuled in a zeolite cage	pH = 3 Peroxydisulfate	0.70 mg g^−1^	[128]
MOF-Fe	PDA-modified Fe-containing MOF	pH = 7 Persulfate	1.50 mg g^−1^	[129]
CoFe_2_O_4_@NPC	N-doped porous carbon coated bimetallic zeolitic imidazolate framework	pH = 6 Persulfate	11.33 mg g^−1^	[130]
Fe_3_O_4_@Activated carbon	Iron-based oxide dispersed on carbon material	pH = 3 Persulfate	2.50 mg g^−1^	[131]
Pyrite@zeolite	S-Fe interaction	pH = 7 PAA	1.62 mg g^−1^	[28]

## Data Availability

No new data were created or analyzed in this study. Data sharing is not applicable to this article.
